# Estimating the impact of a novel drug regimen for treatment of tuberculosis: a modeling analysis of projected patient outcomes and epidemiological considerations

**DOI:** 10.1186/s12879-019-4429-x

**Published:** 2019-09-09

**Authors:** Emily A. Kendall, Shelly Malhotra, Sarah Cook-Scalise, Claudia M. Denkinger, David W. Dowdy

**Affiliations:** 1 Division of Infectious Diseases and Center for Tuberculosis Research, Johns Hopkins University School of Medicine, CRB2 Room 106, 1550 Orleans St, Baltimore, MD 21287 USA; 2 Global Alliance for TB Drug Development, New York, NY USA; 3 International AIDS Vaccine Initiative, New York, NY USA; 4 Division of Tropical Medicine, Center of Infectious Disease, Heidelberg University, Heidelberg, Germany; 5 Tuberculosis Programme, FIND, Geneva, Switzerland; 6 Department of Epidemiology, Johns Hopkins Bloomberg School of Public Health, Baltimore, MD USA

**Keywords:** Tuberculosis, Treatment, Regimen selection, Drug resistance, Novel regimens, Clinical outcomes, Modeling

## Abstract

**Background:**

Regimens that could treat both rifampin-resistant (RR) and rifampin-susceptible tuberculosis (TB) while shortening the treatment duration have reached late-stage clinical trials. Decisions about whether and how to implement such regimens will require an understanding of their likely clinical impact and how this impact depends on local epidemiology and implementation strategy.

**Methods:**

A Markov state-transition model of 100,000 representative South African adults with TB was used to simulate implementation of the regimen BPaMZ (bedaquiline, pretomanid, moxifloxacin, and pyrazinamide), either for RR-TB only or universally for all patients. Patient outcomes, including cure rates, time with active TB, and time on treatment, were compared to outcomes under current care. Sensitivity analyses varied the drug-resistance epidemiology, rifampin susceptibility testing practices, and regimen efficacy.

**Results:**

Using BPaMZ exclusively for RR-TB increased the proportion of all RR-TB that was cured by initial treatment from 60 ± 1% to 67 ± 1%. Expanding use of BPaMZ to all patients increased cure of RR-TB to 89 ± 1% and cure of all TB from 87.3 ± 0.1% to 89.5 ± 0.1%, while shortening treatment by 1.9 months/person. In sensitivity analyses, reducing the coverage of rifampin susceptibility testing resulted in lower projected proportions of patients cured under all regimen scenarios (current care, RR-only BPaMZ, and universal BPaMZ), compared to the proportions projected using South Africa’s high coverage; however, this reduced coverage resulted in greater expected incremental benefits of universal BPaMZ implementation, both when compared to RR-only BPaMZ implementation and when compared to to current care under the same low rifampin susceptibility testing coverage. In settings with higher RR-TB prevalence, the benefits of BPaMZ were magnified both for RR-specific and universal BPaMZ implementation.

**Conclusions:**

Novel regimens such as BPaMZ could improve RR-TB outcomes and shorten treatment for all patients, particularly with universal use. Decision-makers weighing early options for implementing such regimens at scale will want to consider the expected impact on patient outcomes and on the burden of treatment in their local context.

**Electronic supplementary material:**

The online version of this article (10.1186/s12879-019-4429-x) contains supplementary material, which is available to authorized users.

## Background

Annually, ten million people develop tuberculosis (TB), and more than one million die of TB [1]. Treatment remains arduous, and relapse rates after first-line treatment exceed 5% [2]. For TB that is rifampin-resistant (RR) or multidrug-resistant, the necessities of an additional drug-resistant detection step and of treatment lasting 9 to 18 months pose even greater challenges. Shorter and more universally effective regimens which treat both rifampin-susceptible (RS) and RR-TB in 6 months or less could be transformative [3].

One potential such regimen in late-stage clinical development is BPaMZ, which combines the novel TB drugs bedaquiline (B) and pretomanid (Pa) with the first-line TB drug pyrazinamide (Z) and the third-generation fluoroquinolone moxifloxacin (M). Data from the NC-005 Phase 2B human study suggest that at 8 weeks, even subsets of the BPaMZ regimen – namely, BPaZ for drug-susceptible TB, or BPaMZ in patients with pyrazinamide-resistant strains of multidrug-resistant TB – surpassed the performance of the current standard regimen (HRZE [isoniazid, rifampin, pyrazinamide, and ethambutol]) in pan-susceptible TB [4]. In murine models, BPaMZ demonstrated powerful sterilizing activity as well, and even BPa alone rivaled the efficacy of HRZE [5]. A phase 2c/3 trial of the BPaMZ regimen (SimpliciTB) is underway, evaluating its potential to both shorten treatment to 4 months for patients with drug-susceptible TB and also effectively treat (as a 6-month regimen, because of their higher risk of associated resistance to pyrazinamide) patients with rifampin- or multidrug-resistant TB [6].

If this regimen continues to prove successful, policy-makers will need to understand its expected impact on clinical outcomes in order to implement it effectively and allocate resources appropriately. A key decision is whether to adopt the regimen for patients with rifampin-resistant TB, rifampin-susceptible, or both, and to what extent this decision depends upon local epidemiology.

We therefore constructed a model of clinical outcomes among a hypothetical cohort of people with TB in South Africa. Our primary objective was to quantify the potential epidemiological benefit and adverse consequences (e.g., emergence of drug resistance) associated with this regimen.

## Methods

### Simulated cohort and Markov model

**Fig. 1:**
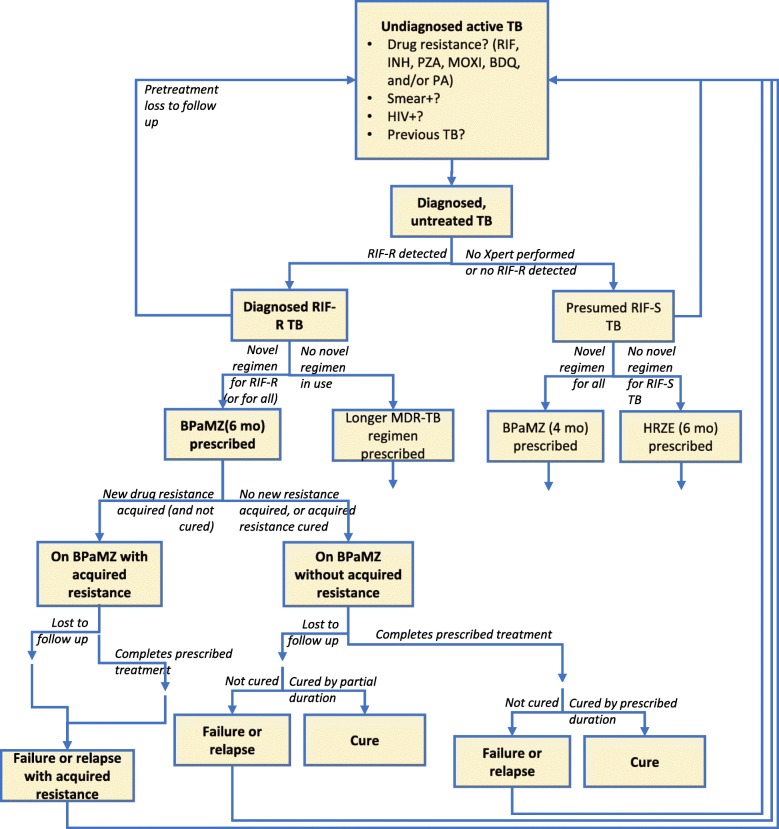
Model diagram. The full pathway of treatment and beyond is shown here only for RR-TB receiving the novel regimen, but other pathways proceed similarly. Transition probabilities depend on characteristics of the individual patient and on the BPaMZ implementation scenario modeled. Probability of cure depends on the drugs in the regimen prescribed, the initial drug resistance, and the duration of treatment completed. Acquired resistance, by definition, means the patient will not be cured; the probability of acquired resistance is accounted for in the overall probability of failure or relapse for each possible combination of patient and treatment course. Failure or relapse is split into failure (which immediately returns to active TB) and relapse (which becomes active after a short delay). Loss to follow up is modeled as a constant hazard during treatment, with cumulative risk thus depending on the treatment duration. Death, not shown, also may occur from any state, with mortality being increased among patients with HIV and/or TB

We developed a Markov model to simulate the course of TB disease for a cohort of people with pulmonary TB (Fig. 1). Using this model, we followed individuals under different algorithms for BPaMZ regimen use and different underlying epidemiologic and regimen-efficacy assumptions, considering outcomes of cure, time with TB, prevalence and acquisition of drug resistance, and drug utilization.

Individuals were characterized according to TB status, time since TB onset, previous TB treatment, HIV infection status, smear status at time of diagnosis, and susceptibility to each of six different drug classes. The initial characteristics within the cohort reflected their distribution and correlations among notified TB in South Africa, from data sources including notifications to the national program [1] and drug resistance surveys [7, 8] (Table 1, Additional file 1: Table S1).

**Table 1:** Select model parameters

Parameter	Estimate, South Africa	Range in sensitivity analysis	References and notes
Fraction of TB cases previously treated for TB	10%	8–13%	[9]
Fraction of TB cases with HIV	60%	54–66%	[1]
Baseline prevalence of RR, new cases	3.4%	2.5–4.3%	[1]
Baseline prevalence of RR, cases previously treated for TB	7.1%	4.8–9.5%	[1]
Prevalence of pyrazinamide resistance, if RR	44%	33–55%	[7]
Prevalence of pyrazinamide resistance, if RS	1.3%	0.8–2.0%	[7]
Prevalence of any moxifloxacin resistance, if RR	9.5%	4–18%	[7, 10]
Prevalence of high-level moxifloxacin resistance, if RR	5.9%	2–12%	[7, 10]
Prevalence of any moxifloxacin resistance, if RS	0.4%	0–0.9%	[7, 10]
Mean time from TB onset to TB diagnosis	9 months	6–15	Incidence:prevalence ratio estimates [1]
Pretreatment loss to follow up	10%	5–20%	[11]
Monthly loss to follow up during treatment	1%	0.8–2%	[12]
Monthly TB mortality, untreated active TB	2.1%	2.3–2.8%	[1, 13]
Present-day Xpert MTB/RIF coverage, new patients	70%	60–80%	[1] with projected increase to 2019
Present-day Xpert MTB/RIF coverage, patients previously treated for TB	75%	60–90%	[1] with projected increase to 2019
Relapse after six months HRZE or four months of BPaMZ (assuming drug susceptibility)^a^	6.3%	2–12%	[4, 14–16]; See Additional File 1.
Odds ratio of cure from moxifloxacin-containing regimen, low-level versus high-level moxifloxacin resistance	1.7	1.3–2.2	[17], based on levofloxacin vs ofloxacin when ofloxacin resistant
Risk of acquired RR after HRZE ^b^	0.005	0.002–0.15	[18]
Risks of acquired B, Pa, or M after BPaMZ ^b^	0.002	0–0.01	Assumed
Risk of acquired moxifloxacin resistance after conventional multidrug-resistant TB regimen	0.04	0.005–0.08	[19]

TB = tuberculosis, RS = rifampin susceptible, RR = rifampin resistant, Z = pyrazinamide, M = moxifloxacin. B = bedaquiline, Pa = pretomanid, HRZE = standard first-line regimen of isoniazid, rifampin, pyrazinamide, ethambutol.

^a^ The probability of successful treatment is reduced when resistance is present to one or more drugs in the regimen prescribed, or when duration is changed (shortened due to loss to follow up, or extended to six months for patients with RR-TB receiving BPaMZ), as shown in part b of the Table

^b^ Parameter value shown is the risk if initially susceptible to R and Z (HRZE), or to B, Pa, and M (BPaMZ). Risk of acquired resistance to remaining drugs is increased for *M. tuberculosis* strains already resistant to one or more of these drugs in the regimen used; see Additional File 1 for details

Cases were followed from the onset of a new or recurrent TB episode. Modeled events included TB diagnosis, Xpert-based rifampin DST where available, regimen selection, treatment (modeling duration as the number of months prescribed, or fewer if loss to follow up occurred), and treatment outcomes of either cure, or failure or relapse (with or without newly acquired drug resistance). Diagnosis and treatment could occur up to four times if initial diagnosis did not lead to curative treatment. Parameters are listed in Table 1 and in Additional file 1: Table S2.

### Projecting individual treatment outcomes

The probabilities of achieving cure, and of acquiring drug resistance if not cured, each depended on the combined effects of all prescribed drugs to which the patient’s disease was susceptible. Probability of cure additionally depended on the duration of treatment completed.

In the absence of data on clinical cure after BPaMZ, probabilities of cure were extrapolated from probabilities of culture conversion at 8 weeks in clinical trials [4, 20], using a regression model derived from historical trial data [21]. We additionally imposed the assumption that for drug-susceptible TB, 4 months of BPaMZ had a relapse rate equivalent to 6 months of HRZE – consistent with culture conversion data and current trial design [4, 6]. Details are provided in Additional file 1, including Tables S3 and S4, with select cure probabilities summarized in Table 2.

**Table 2:** Selected probabilities of durable cure, by active drugs and duration of treatment^a^

Active drugs in prescribed regimen	4 months	6 months	18 months
HR(ZE) ^b^	86.0%^c^	94.4%	Not applicable
R(ZE) ^b^	58.0%^c^	83.3%	Not applicable
BPaMZ	93.7%	97.6%	Not applicable
BPamZ ^d^	91.6%	96.8%	Not applicable
BPaM	89.5%	95.9%	Not applicable
BPaZ	86.5%	94.6%	Not applicable
BPam ^d^	70.2%	89.4%	Not applicable
Conventional multidrug-resistant TB regimen with full fluoroquinolone activity [22, 23]	20.0%^c^	40.2%^c^	91.3%
Conventional multidrug-resistant TB regimen in presence of fluoroquinolone resistance [22, 23]	20.0%^c^	20.0%^c^	79.1%

^a^ Modeled as a function of two-month culture conversion and time on treatment, for the set of drugs in the prescribed regimen to which the patient’s TB strain is susceptible. Details in Additional file 1

^b^ Outcomes of HRZE are affected explicitly by isoniazid and/or rifampin resistance, but because data for the HRZE regimen come from studies that did not test for pyrazinamide or ethambutol resistance, outcomes are weighted averages reflecting the distribution of pyrazinamide and ethambutol resistance within each patient subpopulation

^c^ Durations of 4 months for HRZE, and of 4 or 6 month for conventional MDR regimens, are shown for comparison but are not prescribed and are used within the model only if patients are lost to follow up at these time points

^d^ “m” represents a moxifloxacin-containing regimen used to treat a TB strain that has low-level moxifloxacin resistance

Although BPaMZ does not contain rifampin, the currently proposed strategy for assignment of BPaMZ treatment duration leverages the known association between resistance to rifampin (for which accurate, rapid DST is increasingly performed) and resistance to pyrazinamide as a component of BPaMZ. [6, 7]. Accordingly, we assumed that BPaMZ would be prescribed for 6 months for patients known to have RR-TB, and for 4 months otherwise. This is a pragmatic strategy that acknowledges that fluoroquinolone resistance testing is not performed widely.

For parameters describing the risk of acquiring drug resistance during treatment, our primary analysis assumed that when pan-susceptible TB was treated with BPaMZ, the combined risk of acquiring resistance to any of the included drugs (except for pyrazinamide) equaled the risk of acquiring resistance to rifampin when treated with HRZE. Remaining agnostic to which drug resistance would develop first, we divided the risk of acquired resistance equally between moxifloxacin, bedaquiline, and pretomanid. Pre-existing resistance increased the risk of acquiring resistance to additional drugs (Additional file 1: Tables S5 and S6).

### Implementation scenarios

We considered the impact of BPaMZ in South Africa if all patients with TB were eligible for the new regimen (“Universal BPaMZ”), or if only those with known RR-TB were eligible (“RR-only BPaMZ”), comparing each to outcomes under “Current Care”.

In scenario analyses extrapolating settings other than South Africa, we also considered how projected impact changed with:

- Lower Xpert coverage (rifampin DST for only 10% of new patients and 37.5% [half the current level in South Africa] of retreatment patients), and/or
- Higher RR prevalence (3x higher odds of RR).
- Higher prevalence of moxifloxacin and pyrazinamide resistance (3-fold higher odds of each)

In sensitivity analyses, we evaluated how projections of BPaMZ impact were affected by:

- Lower BPaMZ efficacy (requiring 5 months of BPaMZ, rather than four, to achieve the efficacy of 6 months of HRZE)
- An improved multidrug-resistant TB standard of care (a 12-month regimen achieving outcomes similar to HRZE), reflecting ongoing improvements in the efficacy, outcomes, and duration of multidrug-resistant TB treatment [24, 25]

For outcomes specific to potential acquisition or transmission of drug-resistant TB, we also evaluated the effects of:

- Higher risks of BPaMZ resistance acquisition (including minimum 1% risk of acquired resistance to each of moxifloxacin, bedaquiline, and pretomanid), or
- A nonzero (2%) initial prevalence of bedaquiline resistance in the cohort, for example reflecting spontaneously-occurring resistance or clofazimine cross-resistance [26, 27].

Additional sensitivity analysis details are provided in Additional File 1.

### Reporting of outcomes

We first repeatedly simulated disease courses for each possible set of baseline individual-patient characteristics (minimum 5000 times each; 50,000 times each for patient types comprising more than 5% of the cohort). From those simulated courses, we randomly sampled with replacement, weighting by the expected frequency of each set of patient characteristics within a South African cohort. We report results as a mean ± standard deviation across 50 such cohorts of 100,000 people with TB.

Rather than a single combined utility, we measure and report the impact of regimen selection on multiple outcomes, including cure, time with active TB or active drug-resistant TB, and months of TB treatment administered. TB cure, in particular, is also evaluated in several ways: We first consider the proportion of all individuals with TB who were cured within one diagnosis and treatment attempt, taking into account death before treatment, or initial loss to follow up, in the denominator (henceforth named “all individuals with TB”). We also consider the proportion cured among those individuals who were treated for TB (those who initiated a treatment regimen, independent of whether the regimen selected was appropriate for their TB strain or whether they completed the full regimen; henceforth named “patients treated”). Furthermore, we consider the proportion achieving within a certain number of months of TB onset, and the proportion ultimately achieving cure after multiple rounds of diagnosis and treatment.

## Results

### BPaMZ and cure of RS- and RR-TB in South Africa

**Fig. 2:**
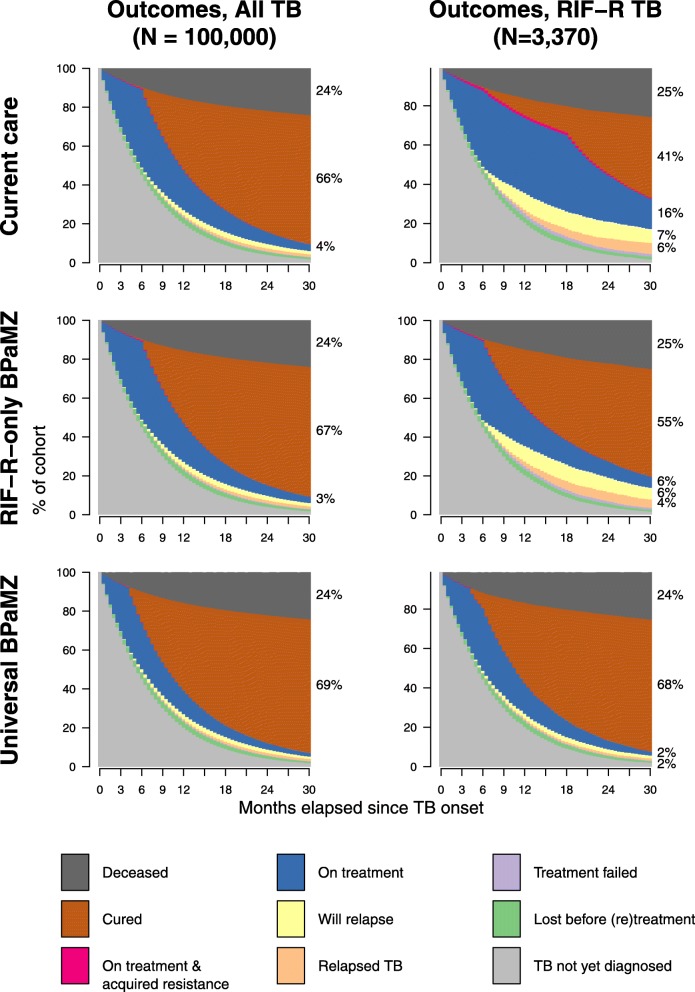
Simulated impact of BPaMZ regimen on status of South African TB cohort and RR sub-cohort over time. Scenarios modeled are: (**a**) standard of care baseline (including conventional DR-TB regimens for those found to have RR-TB, and HRZE for all others; top row), (**b**) introduction of the novel regimen for patients known to have RR-TB (middle row), and (**c**) introduction of the novel regimen for all patients (with duration dependent on the DST result if rifampin DST is performed; bottom row). The percentages along the right edge of each panel show the fraction of the cohort in each state 30 months after onset of TB (with fractions < 2% not labeled)

The impact of the BPaMZ regimen among all individuals with TB, and among those all individuals with RR-TB, is shown in Fig. 2 (additional outcomes in Additional file 1: Table S7). Under Current Care, a single round of treatment cured only 45.0 ± 0.7% of all individuals with RR-TB (including those never diagnosed or treated, and those whose resistance was not diagnosed), for reasons that include TB under-diagnosis, mortality before and after TB diagnosis, loss to follow up before and during treatment, failure and relapse after RR-TB treatment, and failure and relapse after HRZE treatment of undiagnosed RR-TB. BPaMZ use for only RR-TB increased this proportion cured to 50.3 ± 0.8% of all individuals with RR-TB. Limiting analysis to RR-TB patients treated (i.e., to individuals with RR-TB who initiated any TB treatment), the proportion cured was 60.0 ± 0.9% under Current Care and 67.1 ± 0.9% when BPaMZ was used for recognized RR-TB.

Using BPaMZ universally provided additional benefit, much of which also accrued to patients with RR-TB. The universal indication facilitated more effective treatment for the 48% of TB with RR that went undetected (due to non-bacteriologic TB diagnoses, incomplete Xpert coverage, or imperfect Xpert sensitivity). It thus further increased RR-TB cure after the first round of treatment, to 66.7 ± 0.6% of all individuals with RR-TB and 88.8 ± 0.6% of RR-TB patients treated with any TB treatment.

For individuals with RS-TB, treatment with Current Care achieved cure for 66.2 ± 0.2% of all individuals with RS-TB and 88.4 ± 0.1% of RS-TB patients treated. Universal use of BPaMZ increased these probabilities of RS-TB cure by only 1%, but it maintained high cure rates – 67.0 ± 0.2% of all individuals with RS-TB, or 89.5 ± 0.1% of RS-TB patients treated – with the advantage of a shorter 4-month treatment duration.

Considering all individuals with TB and up to four rounds of treatment, the proportion ultimately cured of TB increased slightly from 77.8 ± 0.1% with Current Care to 78.9 ± 0.1% with Universal BPaMZ.

### Impact of BPaMZ regimen on treatment duration and medication use

Comparing Universal BPaMZ to Current Care, the average cumulative treatment time fell from 5.4 months to 3.5 months per individual in the cohort (Additional file 1: Table S7). These totals include those never treated, those lost to follow up after partial treatment courses, and those requiring retreatments, among both RS- and RR-TB.

Per 1000 individuals with TB followed for up to four rounds of diagnosis and attempted treatment, a switch from Current Care to RR-only use of BPaMZ eliminated total 27 ± 1 months of treatment (with multidrug-resistant TB regimens) and replaced an additional 14 ± 1 months of conventional multidrug-resistant TB treatment with the same number of months of the BPaMZ regimen. Using BPaMZ universally, as opposed to only for RR-TB, further eliminated 170 ± 1 months of treatment (with the additional eliminated treatment months being months of the HRZE regimen) and replaced an additional 333 ± 1 months of HRZE with the same number of months of BPaMZ.

### Expected impact on transmission potential and drug resistance

Compared to Current Care, Universal BPaMZ reduced the total time with active (and potentially-infectious) TB by 6.4 ± 0.6 months per person (a 41 ± 3% reduction) among those who had RR-TB at the start of the model, and by 0.33 ± 0.05 months per case (a 3.5 ± 0.6% reduction) in the overall TB cohort.

**Fig. 3:**
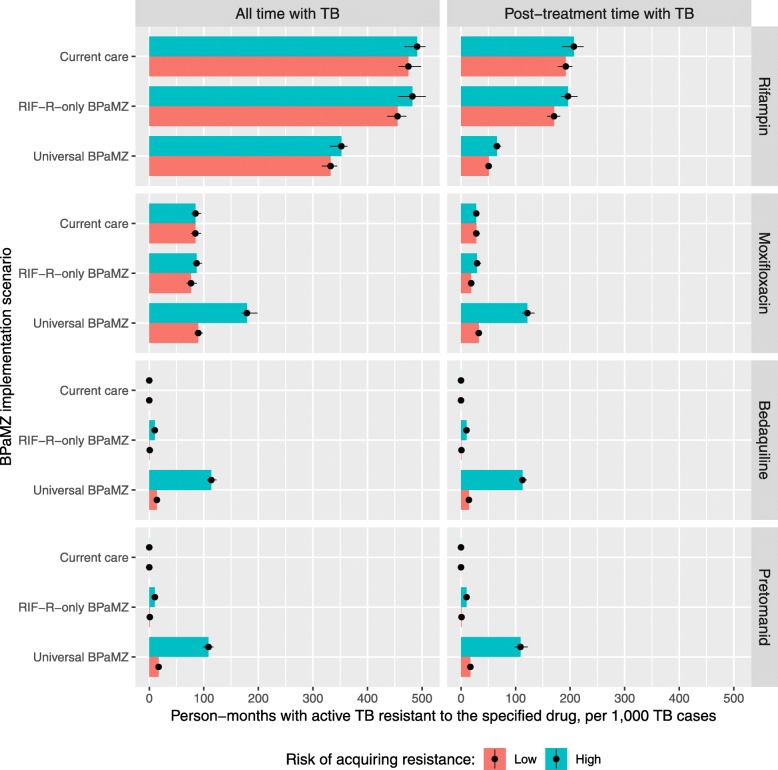
Impact of BPaMZ regimen use and barrier to resistance on potentially infectious person-time. Total time with drug-resistant TB (left column) includes the time prior to the first treatment within the model, while the right column shows time with active drug-resistant TB after an individual has begun treatment at least once within the model – that is, time and potential transmission that better treatment might have prevented. For comparison, in the Current Care scenario, total time with TB (with or without drug resistance) was 8900 person-months overall and 1400 person-months after a treatment attempt, per 100 TB cases. Acquired resistance risk parameters increase risks of moxifloxacin, bedaquiline, and pretomanid resistance during BPaMZ, and also of isoniazid resistance during HRZE

The use of BPaMZ resulted in an increase in bedaquiline and pretomanid resistance, and also had potential to increase moxifloxacin resistance when used as a universal regimen (Fig. 3). However, the amount of new drug resistance created could be small compared to the reduction in RR-TB, when measured in terms of potentially infectious person-time within a single TB cohort. Under our initial assumption of a relatively low risk of drug resistance acquisition during treatment of initially-pan-susceptible TB with BPaMZ, the switch from Current Care to Universal BPaMZ eliminated 14 ± 1 months of active RR-TB, while adding a combined 4 ± 1 months of active moxifloxacin-resistant, bedaquiline-resistant, and/or pretomanid-resistant TB. In the sensitivity analysis with approximately five-fold higher estimated risks of resistance acquisition during BPaMZ treatment, the aggregate increase in time with moxifloxacin-resistant, bedaquiline-resistant, and/or pretomanid-resistant TB exceeded the reduction in time with RR-TB by a factor of two: 32 ± 1 months of active MXR-, bedaquiline-, or pretomanid-resistant TB added, in exchange for approximately the same 14 ± 1 months of RR-TB averted (Fig. 3). Finally, under the assumption of a nonzero (2%) initial prevalence of bedaquiline resistance within the cohort (and low risk of resistance acquisition when initially pan-susceptible), overall bedaquiline-resistance transmission increased. However, because cure rates remained relatively high at 80.6 ± 0.1% for bedaquiline-resistant TB (including polydrug resistant TB), Universal BPaMZ caused relatively small increases in the expected transmission of bedaquiline-, moxifloxacin-, and pretomanid-resistant TB relative to expected transmission of bedaquiline-resistant and moxifloxacin-resistant TB under current care (Additional file 1: Figure. S1).

### Dependence of BPaMZ impact on local RR-TB epidemiology and detection

**Fig. 4:**
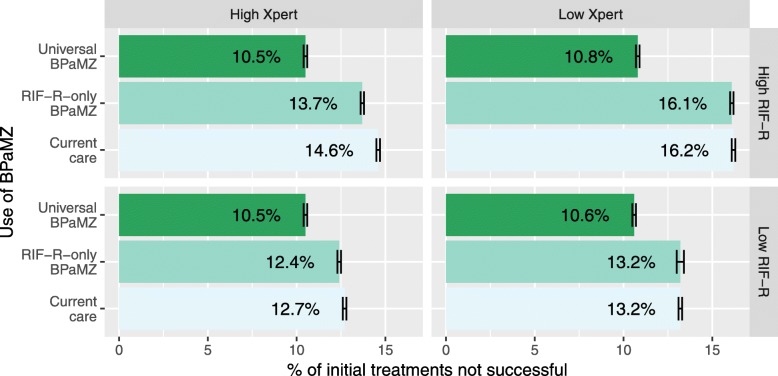
BPaMZ treatment outcomes in different scenarios of local RR prevalence and rifampin DST (Xpert) coverage. “High Xpert” coverage reaches 70% of new and 75% of previously-treated patients, and “Low Xpert” coverage reaches 10% of new and 37.5% of retreatment patients. “Low RIF-R” prevalence is RR in 3.4% of new and 7.1% of previously-treated TB (3.8% overall), and “High RIF-R” prevalence increases the odds of RR-TB three-fold, to RR prevalence of 9.6% of new and 18.7% of retreatment patients (10.5% overall). The lower left panel thus represents the base model of present-day South Africa. Error bars show the standard deviation over repeated simulations of each scenario with cohorts of 100,000 patients in each setting

In the South African setting modeled, the combined proportion of new and retreatment TB cases with RR was below the global average, and rifampin DST coverage was high. Varying these conditions had important effects on the expected impact of BPaMZ, and on the advantages of Universal versus RR-only BPaMZ use (Fig. 4).

First, increasing the prevalence of RR from 3.8% to near 10.5%, while maintaining high Xpert coverage, maintained the expectation of a high cure rate (89.5 ± 0.1%) for Universal BPaMZ (Fig. 4). At the same time, this higher RR prevalence (combined with low DR-TB treatment success rates under Current Care) increased the extent to which Universal BPaMZ could improve upon the status quo (a 4.1 ± 0.1% increase in overall cure rate versus Current Care, compared to a 2.2 ± 0.1% increase with lower RR prevalence). Higher RR prevalence also increased the fraction of BPaMZ’s total impact that could be achieved through use of BPaMZ for RR-TB alone, although Universal BPaMZ use still accounted for most (approximately 85%) of BPaMZ’s potential to increase cure.

If Xpert coverage fell (reducing rifampin DST availability from 70 to 10% of new cases and from 75 to 37.5% of retreatment cases), then the impact of RR-only BPaMZ use on overall TB cure rates became negligible (remaining at 86.8 ± 0.2% cure with low RR prevalence, and 85.9 ± 0.1% with high RR prevalence, Fig. 4). Meanwhile, the benefits of Universal BPaMZ over Current Care grew, because the universal regimen benefitted TB patients with undetected RR. The benefits of Universal BPaMZ over Current Care were maximized in a setting where Xpert coverage was low and RR prevalence simultaneously high (where it increased cure by 5% from 83.9 ± 0.1% to 89.2 ± 0.1%, versus a 2% increase in the base model), even though such a scenario resulted in slightly more patients having poor BPaMZ treatment outcomes than in other settings (10.8 ± 0.1% of patients, compared to 10.5 ± 0.1% in the base model, as a result of more patients with moxifloxacin-resistant and/or pyrazinamide-resistant TB receiving only 4 months of BPaMZ).

### Additional scenario and sensitivity analyses

We repeated the analysis with three-fold higher odds of moxifloxacin and pyrazinamide resistance – a change which increased the prevalence of moxifloxacin resistance and pyrazinamide resistance in the cohort to 4 and 1%, respectively, among RIF-S TB, and to 70 and 25% among RR-TB, leading to resistance resembling the former Soviet Union region in the model scenario with high RR prevalence as well. This change reduced BPaMZ cure rates by < 0.5% in the reference model, and by 0.5 to 1% in settings with high RR prevalence and/or low Xpert coverage (Additional file 1: Figure. S3).

With higher estimated probabilities of acquiring moxifloxacin, bedaquiline, or pretomanid resistance during BPaMZ treatment, initial cure rates remained unchanged, but among those not cured by initial treatment, the prevalence of drug resistance increased, leading to worse outcomes during retreatment. Thus, the proportion of all individuals with TB who were cured within two rounds of attempted treatment with Universal BPaMZ fell slightly from 77.1 ± 0.1% to 76.9 ± 0.1%; among patients with RR-TB, this proportion fell from 76.6 ± 0.7% to 73.2 ± 0.8%. Considering only those who were retreated after not being cured by initial treatment, higher estimates of acquired resistance reduced the proportion cured from 88.9 ± 0.5% to 85.8 ± 0.3% overall, and from 86.4 ± 1.3% to 65.6 ± 2.4% for initial RR-TB (where pyrazinamide resistance or moxifloxacin resistance were a contributor in most initial failures to cure).

Lowering BPaMZ efficacy (to 91% non-relapsing cure after 4 months if pan-susceptible – a value potentially still within clinical trial noninferiority margins) lowered the expected Universal BPaMZ cure rates in South Africa from 89.5 ± 0.1% to 87.3 ± 0.1% and prevented BPaMZ from improving treatment outcomes over Current Care in most settings (Additional file 1: Figure. S4).

Finally, improving the standard of care for RR-TB prior to BPaMZ introduction reduced the benefits of BPaMZ among patient with RR-TB, but maintained some benefit: among RR-TB patients who received any treatment, the percentage cured with RR-only implementation of BPaMZ rose by only 3% (from 64 to 67%) relative to the improved RR-TB standard of care, compared to the 7% increase with BPaMZ relative to Current Care (from 60 to 67%) in our original analysis. Because patients receiving the drug-resistant-TB-specific regimen accounted for a small fraction of total poor outcomes, BPaMZ’s impact on overall cure rates was minimally affected (Additional file 1: Figure. S5).

## Discussion

If the outstanding performance of the BPaMZ regimen in preliminary studies is confirmed in larger-scale trials, it could offer an important advance by shortening treatment durations using a single drug combination for nearly all patients with TB. While patients with RR-TB would derive the greatest benefits, using such a regimen universally (for RS- and well as RR-TB) could offer important additional benefits for both groups of patients: for patients with RS-TB, it would shorten the treatment duration, while for patients with RR-TB that might be missed by DST practices, it could substantially increase cure. Modeling a South African TB cohort, we have estimated that implementing the BPaMZ regimen universally (with stratification of treatment duration based on rifampin susceptibility) could simultaneously increase the percentage of patients with RR-TB who are cured from 60% to nearly 90%, maintain nearly 90% cure among patients with RS-TB, reduce treatment duration by 2 months or more per patient, and reduce infectious person-time by 3% (RS-TB) to 50% (RR-TB). The potential impact of an effective universal regimen is even greater in settings of higher RR prevalence or lower DST coverage.

As BPaMZ or similar regimens become available, TB programs and health systems will need to choose whether to implement such regimens, and whether to restrict their use to patients with RR-TB. Our model shows that the impact of BPaMZ as an RR-only TB treatment regimen depends heavily on the extent to which RR-TB is consistently detected. For settings with moderate or high RR prevalence that cannot achieve extremely high rates of RR, regimen universalization is expected to increase the impact on cure several-fold. Additional advantages of universal implementation would include shorter treatment duration and streamlined care delivery, with benefits for patients and providers. Affordability evaluations will need to consider both the potentially higher costs of replacing low-cost HRZE with novel drugs, and the possible reductions in costs of health care delivery [28].

Acquired resistance and resistance transmission are a potential concern with use of any new drug combination. Our analysis suggests that, although use of a bedaquiline- and pretomanid- containing regimen is expected to create some resistance to these drugs and will require a resistance-management strategy, this is not a compelling reason to avoid universal use in the short term as long as current efficacy estimates for BPaMZ are borne out. For example, even if probabilities of acquiring bedaquiline or pretomanid resistance turn out to be relatively high, initial increases in bedaquiline-resistant or pretomanid-resistant TB transmission could be counterbalanced by reduced RR-TB transmission; in addition, most acquired bedaquiline- and pretomanid resistance would occur in RS-TB strains for which other treatment options would still exist as long as the bedaquiline resistance were identified [29]. Our conclusions resemble those of a previous analysis which favored use of bedaquiline for all multidrug-resistant TB rather than only extensively-drug-resistant TB despite the increase in acquired bedaquiline resistance [30]. However, over several years and several cycles of transmission, compounding novel-drug resistance could pose a barrier to universal use of this novel regimen, with a moderately low barrier to resistance potentially resulting in novel-drug resistance in up to 5% of TB cases after 5 years and more than 10% of TB cases after 50 years under pessimistic assumptions and drug resistance transmission [31]. Our results therefore highlight the need to better quantify risks of spontaneously-occurring [26] and acquired resistance to these drugs, and to take appropriate actions to detect and contain resistance. Initial steps to preserve and maximize regimen usefulness include drug-resistance surveillance at the population level, and patient-level identification of risk factors such as previous treatment with BPaMZ (for example, for RR-TB in our model, BPaMZ cure rates were 3 to 30% lower in retreatment than initial treatment, depending on the estimated risks of BPaMZ resistance acquisition) or treatment with other drugs with potential cross-resistance (e.g., delamanid or clofazimine) [27, 32]. Acquisition of bedaquiline or pretomanid resistance would not mean we must avoid using these drugs, but it would indicate an urgent need to develop strong drug resistance surveillance systems and rapid DST.

Our findings should be interpreted in light of certain limitations. First, because no human relapse data yet exist for BPaMZ, projected cure rates must be extrapolated from animal data, early-phase clinical studies, and clinical experience with other TB drugs. It will be important to glean better estimates and revise projections based on phase 2c/3 study results – and, if the regimen succeeds, to conduct post-approval studies of outcomes in rarer but important patient situations such as moxifloxacin-resistant TB. A second important limitation is the lack of explicit representation of TB transmission in this model, although we do look at potentially infectious person-time as a first order approximation. This approach allowed us to model relationships between assumptions and outcomes (including infectious person-time) more transparently, at the expense of neglecting effects on population-level transmission over time, and allowed us to model a large number of different patient characteristics. We considered TB-related outcomes and time on treatment but not drug side effects or safety monitoring, which will differ between regimens and also should be taken into account in clinical and cost-effectiveness evaluations. Finally, for the current analysis we limited our consideration of DST to the use of RR in selecting eligible patients or optimal treatment durations, but DST for fluoroquinolones or pyrazinamide could also have a useful role alongside such a regimen.

## Conclusions

Novel drugs are reshaping drug-resistant TB treatment and could soon transform the broader TB treatment landscape if ongoing clinical trial evaluations identify regimens that improve upon current first-line treatment. Our modeling results show that if BPaMZ or a similar regimen is effective in trials of long-term cure, it could offer important benefits for TB treatment success rates, TB treatment duration, and streamlined TB regimen selection. If such a regimen were used for RR-TB only, the benefits for those patients who received it could be large compared to continued use of conventional MDR-TB regimens. On a population level, as long as emerging resistance to new drugs is appropriately identified and managed, our analysis suggests that universal use of such a regimen would amplify its benefits for patients both with and without rifampin resistance and across a range of drug-resistance epidemiology.

## Additional file

Additional file 1: Supplemental Material. Contains supplementary methods including a complete description of the model, and supplementary results tables and figures. (DOCX 234 kb)

## Data Availability

The datasets used and analysed during the current study are. available from the corresponding author on reasonable request.

## Abbreviations

BPaMZ: Bedaquiline, pretomanid, moxifloxacin, and pyrazinamide
DST: Drug susceptibility testing
HRZE: The current first line regimen of isoniazid, rifampin, pyrazinamide, and ethambutol
RR: Rifampin resistant
RS: Rifampin susceptible
TB: Tuberculosis
