# Supplementary material for: Estimating the impact of a novel drug regimen for treatment of tuberculosis: a modeling analysis of projected patient outcomes and epidemiological considerations
Source: BMC Infect Dis. 2019 Sep 9;19:794. doi: 10.1186/s12879-019-4429-x (PMC6734288; doi:10.1186/s12879-019-4429-x)
Supplement: Supplementary file 1 — Supplemental Material. Contains supplementary methods including a complete description of the model, and supplementary results tables and figures. (DOCX 234 kb) [file 12879_2019_4429_MOESM1_ESM.docx]

**Supplemental Material:**

**Implementing a novel drug regimen for treatment of tuberculosis: a modeling analysis of patient outcomes and epidemiological considerations**

**Supplemental methods**

*Characteristics of patient cohort:*

The estimated proportion of cases with a TB treatment history at the time that they enter the model is based on the fraction that are new among all notified new or retreatment cases in WHO’s notification data for year 2017.^1^

Resistance was modeled to RIF, INH, PZA, MFX, BDQ, and PMD. All were modeled as dichotomous except for MFX, where we model high and low level resistance, above the clinical cutoff and the critical concentration, respectively.^2^ Prevalence and correlations were estimated as follows:

Prevalence of PZA resistance in South Africa pools drug resistance survey results from two South African sites,^3^ stratifying by RIF susceptibility of those isolates.

Prevalence of MFX resistance in South Africa is also modeled based on estimates from pooled data from the same two sites. Because MFX critical concentrations and clinical breakpoints have been lowered since the MGIT MIC cutoff were selected for those drug resistance surveys, we used other data on the distribution of MFX MICs among clinical MDR-TB isolates characterized by Rigouts et al^4^ to estimate what fraction of isolates in the South African drug resistance surveys would have been resistant to MFX at these lower cutoffs. We also accounted for MIC differences between MICs in MGIT media (used in the drug resistance surveys) versus on LJ media (used by Rigouts et al) by translating LJ MICs down one dilution (e.g. treating an MIC of 0.5 on LJ as equivalent to a 0.25 on MGIT), averaging the results of two estimation approaches. First, of MDR-TB isolates with MIC of 4 µg/ml or above on LJ in the study by Rigouts and colleagues (assumed to represent MICs of 2 µg/ml or above on MGIT), there were 80% more with an MIC of 2 µg/ml on LJ (1 µg/ml or above on MGIT, placing them above the revised clinical breakpoint as well). Applying this ratio to the pooled prevalence MFX-R among RIF-R TB in South Africa at an MIC cutoff of 2.0 µg/ml by MGIT (namely, 2.1%),^3^ we estimated a prevalence of 2.1%*1.8 = 3.8% MFX-R above the clinical breakpoint among all RIF-R TB. In a second approach, we considered that of the isolates with an MIC of 1 or 2 µg/ml on LJ as characterized by Rigouts and colleagues (i.e., isolates which we assumed would have been classified as resistant at 0.5 but not at 2.0 µl/ml on MGIT in the report by Zignol and colleagues^3^), 50% had an MIC of 2 rather than 1, and thus were taken to reflect MFX resistance above the clinical breakpoint. Thus, resistance above the revised clinical breakpoint includes all resistance above the 2.0 ug/ml cutoff and half of that between 0.5 and 2.0 ug/ml, for a total prevalence of 2.1%+0.5*(10.1%-2.1%) = 6.1% MFX-R at a cutoff of 1.0 µg/ml on MGIT. We took a geometric mean of these for a final estimate of 4.8% prevalence of high-level MFX-R among RIF-R.

Because of limited data on the prevalence of MFX-R at MIC cutoffs at of 0.25 versus 0.5, we used the MFX-R prevalence at cutoff of 0.5 µg/ml, 10.1% after pooling isolates from two sites, as a conservative estimate of the prevalence of total MFX-R with MIC at or above the MGIT critical concentration of 0.25 µg/ml. This is likely an underestimate, but not by a large amount since the reported prevalence of levofloxacin and ofloxacin resistance were similar to this value as well.

The same process led to estimates for MFX-R among RIF-S TB in South Africa of 0.3% high-level MFX resistance (above the clinical breakpoint) and 0.4% total MFX resistance.

Ranges used in sensitivity analysis are the broadest of (a) a binomial confidence interval based on number of resistant isolates in the drug resistance surveys, (b) the range in estimates over the sites surveyed, (c) the range between estimation methods.

Prevalence of INH-resistance, stratified by RIF susceptibility and by treatment history among RIF-S TB, was estimated from the national drug resistance survey in South Africa.^5^

The initial prevalence of BDQ and PMD resistance were assumed to be zero for most analyses, but the maximum of 2% BDQ resistance for sensitivity analyses was based on an analysis by Villellas et al,^4^ which showed Rv0678 variants with high BDQ MIC in 8 of 347 MDR and 0.7% of non-MDR isolates.

The association between PZA and MFX resistance was modeled as an odds ratio of PZA-R for isolates with any MFX resistance versus MFX susceptible isolates. A small data set published by Alame-Emane et al^6^ was used to calculated this odds ratio, but we also confirmed that it was consistent with the strength of association among isolates with phenotypic data for both PZA and MFX in the much larger ReseqTB database.

The resulting parameters that define the composition of each cohort are shown in Table S1.

**Table S1: Model parameters describing composition of TB case cohort. Parameters are a proportion of the entire cohort, or of the subpopulation named, unless otherwise specified.**

| Parameter | Estimate for South Africa | Range for sensitivity analysis | References and notes |
| --- | --- | --- | --- |
| Retreatment | 0.1 | 0.08-0.13 | ^1^ |
| RIF-R_in_New | 0.034 | 0.025-0.043 | ^7^ |
| RIF-R_in_Retreatment | 0.071 | 0.048-0.095 | ^7^ |
| PZA-R_in_RIF-S | 0.013 | 0.008-0.02 | ^3^ |
| PZA-R_in_RIF-R | 0.4376 | 0.33-0.552 | ^3^ |
| MFX-R-any_in_RIF-S | 0.004 | 1e-04-0.009 | ^3^ |
| MFX-R-any_in_RIF-R | 0.101 | 0.04-0.18 | ^3^ |
| MFX-R-highlevel_in_RIF-S | 0.003 | 1e-04-0.006 | ^3,8^ |
| MFX-R-highlevel_in_RIF-R | 0.048 | 0.02-0.12 | ^3,8^ |
| INH-R_in_NewRIF-S | 0.0562 | 0.047-0.066 | ^5^ |
| INH-R_in_RetreatmentRIF-S | 0.0681 | 0.052-0.071 | ^5^ |
| INH-R_in_RIF-R | 0.6328 | 0.4459-0.8196 | ^5^ |
| BDQ-R | 0 | 0-0.04 | ^4^ |
| PMD-R | 0 | 0-0.04 |  |
| HIV | 0.6 | 0.54-0.66 | ^7^ |
| Smearpos_in_HIVpos | 0.3187 | 0.2-0.45 | ^9,10^ |
| Smearpos_in_HIVneg | 0.5035 | 0.4-0.64 | ^11^ |
| Odds ratio, PZA-R-if_MFX-R | 2.88 | 1-5 | ^6^ |

*Markov model overview*

Parameters relevant to natural history and clinical management are shown in Table S2.

**Table S2: Model parameters related to TB natural history and clinical management**

| Parameter name | Parameter description | Estimate | Range for sensitivity analysis | References and notes |
| --- | --- | --- | --- | --- |
| Tbdxtime | Average months to TB diagnosis, new cases | 9 | 6-15 | ^7^ |
| Tbdxtime _recurrenceratio | Ratio of time to diagnosis, previously-treated vs new | 1 | 0.5-1.5 | Assumed |
| Unavoidableloss | Pretreatment loss to follow up, proportion | 0.1 | 0.05-0.20 | ^12^ |
| Monthlymortality _TB | Monthly mortality from active TB | 0.021 | 0.012-0.028 | ^7,13^ |
| Monthlymortality _background | Monthly mortality without TB or HIV | 00.0021 | 0.0017-0.0028 | Assumes average 40 year remaining life expectancy among HIV-negative population at risk for TB |
| Monthlymortality _HIV | Monthly mortality from HIV | 0.002 | 0.001-0.004 | Unaids.org, ratio of HIV deaths to HIV prevalence |
| Sens_TB_smearpos | Xpert sensitivity for smear positive TB | 0.99 | 0.98-1 | ^14^ |
| Sens_TB_smearneg | Xpert sensitivity for smear negative TB | 0.63 | 0.54-0.71 | ^14^ |
| Sens_RR | Xpert sensitivity for RR if TB detected | 0.95 | 0.90-0.99 | ^14^ |
| Xpert_current _new | 2019 Xpert MTB/RIF coverage, new TB patients | 0.35 | 0.27-0.60 | ^15^, South Africa, projecting continued scale-up |
| Xpert_current_rerx | 2019 Xpert MTB/RIF coverag, retreatment patients | 0.9 | 0.85-0.95 | ^15^, South Africa, projecting continued scale-up |
| Recurrence_time | Mean months from end of treatment to onset of recurrent TB among those who relapse | 11 | 6-18 | Median time, ^16^ |

Patients are modeled from TB onset. Modeled TB status states include undiagnosed, diagnosed awaiting treatment, lost to follow up before treatment, on treatment with or without acquired resistance, cured, failed, pending relapse, relapsed, or deceased. Each experiences competing hazards of TB diagnosis and mortality until one of these events occurs, and the time is documented. Diagnosis is modeled as occurring at a rate independent of Xpert implementation. If diagnosis occurs, and if the patient is not lost to follow up prior to starting treatment, then DST and regimen selection occur, followed by treatment initiation. Modeling of the treatment course and treatment outcomes are described in detail below. After treatment, for those alive and not yet cured of TB, some who fail have active TB immediately and the remainder relapse after some time. They then undergo repeat diagnosis and treatment, potentially for multiple cycles; the analysis presented here model up to four cycles of diagnosis and attempted treatment. All continue to face hazard of mortality (higher during active TB) throughout the model.

For events with an associated probability or proportion, outcomes for each individual are assigned by independent random draws, and events associated with a rate (mortality, diagnosis, loss to follow up, relapse) are stochastic and follow a Poisson distribution.

*Modeling diagnosis: Drug susceptibility testing and regimen selection*

The probability that Xpert MTB/RIF is performed for RIF DST depends on the scenario modeled (current versus expanded Xpert coverage) and the patient’s treatment history (higher among retreatment patients, for the current Xpert coverage scenarios). TB diagnosis is not modeled as depending on Xpert detection, but detection of resistance requires that Xpert first detect TB (which occurs with a probability that depends on smear status, which in turn is also associated with HIV status). The sensitivity of Xpert for RIF-R is then applied among those patients in whom TB is detected. False positive diagnoses of resistance (imperfect specificity) are not included in this model.

Once any DST results are obtained, a regimen is selected according to the algorithm defined by the implementation scenario. Potential regimens include conventional MDR therapy (“MDR”) in the baseline scenario, modeled as lasting 18 months; conventional first-line therapy for drug-susceptible TB (“HRZE”) for six months; or four or six months of BPaMZ depending on whether RIF-R has been identified and which types of patients are eligible for BPaMZ.

*Modeling treatment: acquisition of drug resistance*

Acquired drug resistance is defined as resistance that is not present in the predominant strain prior to treatment, but that will be present in persistent or recurrent TB after treatment. Drug resistance may develop to RIF, INH, MFX, BDQ, or PMD if these drugs are in the treatment regimen, and the probability of acquired resistance to each drug depends on the combination of active drugs in the regimen. The probability of acquired resistance depends on the initial drug resistance profile and the regimen prescribed, and any acquired drug resistance that will arise is assigned once treatment begins.

To estimate the probabilities of acquired resistance, we begin with parameter estimates based on data for risks of resistance acquisition to RIF, INH, and MFX during treatment with conventional regimens (Table S5). We then make assumptions about the risk of acquired resistance during BPaMZ treatment, assuming the risk to be equal for each of BDQ, PMD, and MFX (when all drugs are fully active at baseline), and selecting this parameter value such that for fully-drug-susceptible TB, the combined risk of acquired resistance to any of MFX, BDQ, or PMD is equal to the risk of acquiring RIF-R during treatment with HRZE. This assumption reflects the anticipated mutual protection that these multiple efficacious drugs provide to one another when treating pan-susceptible TB. We then estimate two additional parameters that function as multipliers: the increase in risk of additional acquired drug resistance when resistance to one drug is already present at the start of treatment, and the further increase when resistance to two drugs is present at baseline. Because PZA likely acts on different *M. tuberculosis* subpopulations in different anatomic compartments than the other drugs, and because data on PZA resistance are limited, we do not model acquisition of PZA resistance, nor do we model PZA activity as protecting against acquisition of resistance to companion drugs. All acquired MFX-R is assumed to be high-level MFX-R. Finally, we assumed that monotherapy resulted in acquired resistance in all who failed treatment or recurred, adjusting the overall probability of acquired resistance to allow for a low rate of spontaneous cure (described further below). Parameters related to acquired drug resistance, and the resulting full array of acquired resistance risks, are shown in Tables S5 and S6.

*Modeling treatment: Probabilities of cure*

During treatment, patients are modeling as facing a constant hazard of mortality (which is reduced to the baseline rate during treatment) and a constant hazard of loss to follow up. If death does not occur, then the total duration of treatment completed is the lesser of the prescribed duration and the time until loss to follow up. The treatment outcome is then determined based on the duration of treatment completed and the activity of the regimen that was prescribed.

We must then estimate the monthly probabilities of cure for each treatment duration and each possible combination of active drugs, including BPaMZ and subsets thereof for which no long-term human data are yet available. We begin with a regimen for which robust clinical data are available: the first-line HRZE regimen for drug-susceptible TB. For the HRZE regimen, we pool data from the control arms of the ReMoxTB, OFLOTUB, and RIFAQUIN trials, to calculate a combined relapse rate of 6.3% among patients with RIF-S TB who completed the 6-month HRZE control regimen and were at risk for recurrence. Because the source data for this model combined patients with pan-susceptible and (rarely) isoniazid mono-resistant TB, we take this 6.3% to be a pooled average, and we then use the pooled prevalence of isoniazid resistance in these three trials (also 6.3%) and an independent estimate that isoniazid monoresistance confers three-fold high risk of failure or relapse after first-line treatment compared to isoniazid susceptible disease,^17^ to estimate separate relapse rates after 6 months of HRZE for rifampicin- and isoniazid-susceptible TB (5.6%) and for isoniazid-monoresistant TB (16.8%). Given limited source data that differentiate patients by PZA or ethambutol susceptibility, we did not stratify HRZE outcomes by PZA or ethambutol resistance status, but assume that the prevalence of these in clinical trial populations was similar to the prevalence in our cohort and that they are thus included with appropriate weights among the INH-S and INH-R populations.

For HRZE treatment durations less than 6 months, we then apply a regression model which uses the log of treatment duration completed and the logit of the proportion achieving 2-month culture conversion as linear predictors of the logit of the proportion who will be cured without recurrence (of those who complete treatment and are at risk for recurrence). ^18^ For the three coefficients of this model, and their uncertainty ranges for sensitivity analysis, we use the estimates of the 2015 model validation and update by Wallis and colleagues,^19^ which are based on fitting to data from multiple large trials of the HRZE regimen.

To estimate the cure rates that would be achieved by the BPaMZ regimen, we start with an assumption which was consistent with the Phase 2B clinical trial data and other existing data, and which would be a necessary condition for regimen approval and availability. Among drug-susceptible clinical trial patients (with TB that is susceptible to RIF, PZA, and MFX) who complete prescribed treatment, we assume that the proportion who relapse after 4 months of BPaMZ would be the same as the proportion relapsing after 6 months or HRZE. Overall rates of cure will therefore be slightly higher for patients with drug-susceptible TB who receive 4 months of BPaMZ rather than 6 months of HRZE, due to less loss to follow up over the shorter treatment duration. Within the same regression model that was used for HRZE, we then estimate the proportion of 2-month culture conversion that would produce these equal relapse rates, verifying that this proportion (98%) matches the available 2-month clinical trial data.^20^ From there, we estimate the relapse rates after BPaMZ durations of less than or greater than 4 months using the regression model described above. Because this model was derived and validated using rifampin-based regimens, we verified correlation of 8-week clinical culture conversion proportions to murine data on bactericidal activity and sterilization/relapse, comparing HRZ(E) to BPaMZ and related drug combinations.^21^

When resistance is present to one or more components of BPaMZ at baseline, we assume that only those drugs with no resistance are active – except in the case of low-level MFX-R, where we assume that MFX retails some activity. We use data from 8-week trials to estimate culture conversion for the following combinations of active drugs: BPaZ (tested among DS-TB patients in NC-005^20^), PaMZ (tested in the earlier NC-002 trial^22^), and BPaM (using results for the BPaMZ regimen among MDR-TB patients later determined to have PZA-R, MFX-S TB^20^). In calculating a single proportion with culture conversion for each drug combination, we pooled data where possible because of small sample sizes: by averaging across all bedaquiline dosing schedules testing and by averaging solid culture results for overnight and spot sputum specimens (but adjusting for the higher average rate of positivity on overnight specimens when no spot specimen results were available). When data did not provide evidence that the efficacies of two regimens were different (PaMZ and BPaZ), we assume they share a single efficacy parameter, and where human culture conversion data were available (BPaZ), we assumed the same efficacy parameter as used for PaMZ and BMZ. When exactly two active drugs out of the BPaMZ regimen were active, we assumed the same outcomes as are achieved by HRZE in cases of INH monoresistance – a conservative assumption given that BPa has performed similarly to HRZ in a murine model.^21^ Finally, for situations in which only a single drug in BPaMZ was active, we defined a parameter for the maximum recurrence rate, to allow for a small degree of “self-cure” with minimal help from treatment; this parameter was based on historical outcomes of RIF-TB TB receiving first-line therapy, and was also used to determine recurrence rates of MDR-TB after HRZE treatment.

For conventional MDR-TB treatment regimens, we did not use 2 month culture conversion to estimate probabilities of cure; instead, recognizing that the timeline of treatment response is slower for these regimens, we used cohort data on probabilities of failure or relapse,^23,24^ then defined an analogous regression curve with a three-fold slower timeline (i.e. using log(duration/3) instead of log duration) to project backward the relationship between treatment duration and probability of cure for those completing fewer than 18 months of treatment. Conventional MDR-TB treatment outcomes were stratified by MFX susceptibility. For rifampin monoresistant TB (which appeared in our South African cohort but not the Southeast Asian cohort used in our primary analyses) treated with HRZE, we similarly assumed a slower timeline, this time by a factor of 1.5, and we estimated the relationship between treatment duration and cure from data on relapse rates after 9 months of HRZE.^25^

For both BPaMZ-related regimens, and conventional MDR treatment, we assumed that in the presence of low-level resistance to MFX (with MIC at or above the critical concentration, but below the clinical breakpoint), MFX would provide a partial contribution to efficacy. We estimated the odds ratio of cure versus relapse, for no MFX versus partial MFX activity, based on the 1.7x adjusted odds ratio of treatment success comparing levofloxacin or moxifloxacin to ofloxacin in the presence of resistance to ofloxacin but not later-generation fluoroquinolones in an individual patient data metaanalysis.^26^

It is important to note that probabilities of cure, failure, or relapse as described in this model are measuring something distinct from treatment success as tracked by TB programs. Cure differs from programmatic treatment success in two directions: On one hand, most patients who relapse would have been counted as a treatment success; while on the other hand, programs report patients with losses to follow up or unknown outcomes, many whom completed at least partial treatment courses that may have been sufficient to achieve cure. Because comparisons will be made to clinical trial data for BPaMZ, we chose to use a similar benchmark for HRZE. Differences between trials and programs will be captured in other aspects of the model, including adherence (dropout prior to 6 months, with worse outcomes) and baseline drug resistance.

The probabilities of relapse defined above are based on data for patients who completed a given duration of treatment with apparent success. To additionally define the corresponding proportion of patients who fail treatment (i.e. those who never have evidence of full treatment response, and who return to active TB immediately when treatment ends), we calculated the ratio of failures to relapses in the REMoxTB, RIFAQUIN, and OFLOTUB trials and applied the same ratio to our cohort for all regimens and treatment durations, calculating the overall proportion with failure in order to maintain this expected ratio of failures per recurrence as fixed of while applying the projected relapse rate as a probability of relapse among those who did not experience failure.

All acquired resistance was modeled as resulting in either failure or relapse (divided according to the same ratio just described). These individuals with acquired relapse are included in the total recurrence and failures rates, and we verified that our parameters were such that total risk of acquired resistance did not exceed our independently-estimated combined risk of failure and relapse. The remainder of those who failed or relapsed, apart from those who acquired resistance, were modeled as continuing with the same drug resistance that they had during their previous TB episode.

Parameters used in defining probabilities of cure versus failure or recurrence are described in Table S3, and the resulting matrix of recurrence probability for all drug combinations and treatment durations is provided in Table S4.

**Table S3: Parameters related to recurrence risks.** See text above for descriptions of references and assumptions used in estimating and applying these parameter values.

| Parameter name | Parameter description | Estimate | Range for sensitivity analyses |
| --- | --- | --- | --- |
| Failures_per_recurrence | Ratio of treatment failures per relapse, for a given regimen and treatment duration | 0.25 | 0.1-1 |
| coef1 | Constant coefficient in regression model of relapse risk: logit(relapse)=2.53-2.50*LN(treatment duration)+0.44*logit(2 month culture positive proportion) | 2.53 | 1.518-3.542 |
| coef2 | Second coefficient in regression model above | 2.5 | 2.05-2.95 |
| coef3 | Third coefficient in regression model above | 0.44 | 0.2464-0.6336 |
| pooled_INH_fraction | Proportion of HRZE-treated patients who had INH-R TB in clinical trial source data | 0.063461 | 0.01-0.15 |
| HRZE_pooled_relapse | Relapse rate, among HRZE-treated patients in clinical trial source data | 0.062789 | 0.02-0.12 |
| INH_multiplier | Increase in odds of relapse after HRZE, INH-resistant versus INH-susceptible TB (all RIF susceptible) | 3 | 1-5 |
| BPaM_cxconv | Proportion achieving 8 week culture conversion if treated with and susceptible to BPaM | 0.94 | 0.89-0.96 |
| BPaZ_cxconv | Proportion achieving 8 week culture conversion if treated with and susceptible to BPaZ; also applied to PaMZ and BMZ | 0.89 | 0.84-0.92 |
| MDR_failrelapse_FQ-S | Proportion of patients with MFX-S MDR-TB with failure or relapse after conventional MDR therapy | 0.109 | 0.07-0.20 |
| MDR_failrelapse_FQ-R | Proportion of patients with MFX-R MDR-TB with failure or relapse after conventional MDR therapy | 0.261 | 0.11-0.40 |
| INHmono_relapse | Proportion of INH-R, RIF-S patients with relapse after 9 months of HRZE | 0.05 | 0.03-0.10 |
| Highrecurrence | Maximum recurrence rate, among those completing treatment with an ineffective regimen | 0.8 | 0.5-1 |
| partialmoxiOR | Increase in odds of cure, partial MFX activity (MFX used in setting of low-level resistance) versus no MFX activity | 1.7 | 1.3-2.2 |

**Table S4: Probabilities of recurrence, as a function of active drugs in regimen and months of treatment completed. These follow from the parameters and assumptions described above.**

| Regimen | Months of Treatment Completed | | | | | | | | | |
| --- | --- | --- | --- | --- | --- | --- | --- | --- | --- | --- |
|  | 1 | 2 | 3 | 4 | 5 | 6 | 9 | 12 | 18 |  |
| HR(ZE) * | 80.0% | 47.9% | 25.0% | 14.0% | 8.5% | 5.6% | NA | NA | NA |  |
| R(ZE) | 80.0% | 80.0% | 75.1% | 42.0% | 25.5% | 16.7% | NA | NA | NA |  |
| H(ZE) | 80.0% | 64.2% | 39.4% | 24.0% | 15.3% | 10.3% | NA | NA | NA |  |
| (ZE) | 80.0% | 80.0% | 80.0% | 80.0% | 80.0% | 80.0% | NA | NA | NA |  |
| MDR, MFX-S | 80.0% | 80.0% | 80.0% | 80.0% | 70.1% | 59.8% | 35.0% | 20.8% | 8.7% |  |
| MDR, MFX-low | 80.0% | 80.0% | 80.0% | 80.0% | 79.2% | 70.7% | 46.7% | 29.9% | 13.4% |  |
| MDR, MFX-R | 80.0% | 80.0% | 80.0% | 80.0% | 80.0% | 80.0% | 59.9% | 42.1% | 20.9% |  |
| BPaMZ | 68.2% | 27.5% | 12.1% | 6.3% | 3.7% | 2.4% | NA | NA | NA |  |
| BPaM | 78.9% | 39.8% | 19.4% | 10.5% | 6.3% | 4.1% | NA | NA | NA |  |
| BPaZ | 80.0% | 46.9% | 24.3% | 13.5% | 8.2% | 5.4% | NA | NA | NA |  |
| BPamZ** | 74.6% | 34.2% | 15.9% | 8.4% | 5.0% | 3.2% | NA | NA | NA |  |
| BMZ | 80.0% | 46.9% | 24.3% | 13.5% | 8.2% | 5.4% | NA | NA | NA |  |
| PaMZ | 80.0% | 46.9% | 24.3% | 13.5% | 8.2% | 5.4% | NA | NA | NA |  |
| BPa | 80.0% | 80.0% | 75.1% | 42.0% | 25.5% | 16.7% | NA | NA | NA |  |
| BPam | 80.0% | 80.0% | 63.9% | 29.8% | 16.8% | 10.6% | NA | NA | NA |  |
| BZ | 80.0% | 80.0% | 75.1% | 42.0% | 25.5% | 16.7% | NA | NA | NA |  |
| BmZ | 80.0% | 80.0% | 63.9% | 29.8% | 16.8% | 10.6% | NA | NA | NA |  |
| BM | 80.0% | 80.0% | 75.1% | 42.0% | 25.5% | 16.7% | NA | NA | NA |  |
| PaZ | 80.0% | 80.0% | 75.1% | 42.0% | 25.5% | 16.7% | NA | NA | NA |  |
| PamZ | 80.0% | 80.0% | 63.9% | 29.8% | 16.8% | 10.6% | NA | NA | NA |  |
| PaM | 80.0% | 80.0% | 75.1% | 42.0% | 25.5% | 16.7% | NA | NA | NA |  |
| MZ | 80.0% | 80.0% | 75.1% | 42.0% | 25.5% | 16.7% | NA | NA | NA |  |
| B | 80.0% | 80.0% | 80.0% | 80.0% | 80.0% | 80.0% | NA | NA | NA |  |
| Bm | 80.0% | 80.0% | 80.0% | 80.0% | 78.8% | 70.2% | NA | NA | NA |  |
| Pa | 80.0% | 80.0% | 80.0% | 80.0% | 80.0% | 80.0% | NA | NA | NA |  |
| Pam | 80.0% | 80.0% | 80.0% | 80.0% | 78.8% | 70.2% | NA | NA | NA |  |
| M | 80.0% | 80.0% | 80.0% | 80.0% | 80.0% | 80.0% | NA | NA | NA |  |
| Z | 80.0% | 80.0% | 80.0% | 80.0% | 80.0% | 80.0% | NA | NA | NA |  |
| mZ | 80.0% | 80.0% | 80.0% | 80.0% | 78.8% | 70.2% | NA | NA | NA |  |
| m | 80.0% | 80.0% | 80.0% | 80.0% | 80.0% | 80.0% | NA | NA | NA |  |
| none | 80.0% | 80.0% | 80.0% | 80.0% | 80.0% | 80.0% | NA | NA | NA |  |

*Because trials have not discriminated HRZE results based on PZA (or ethambutol) susceptibilities, and because PZA-R is rare among RIF-S TB, HRZE recurrence rates are based only on susceptibility to INH and RIF, assuming that the prevalence of PZA-R and ethambutol-R in the model is similar to that in the clinical trials that provided data on HRZE outcomes.

** Lower-case “m” among the active regimen indicates partial activity of MFX.

**Table S5: Parameters related to acquired drug resistance**

| Parameter name | Parameter description | Estimate | Range for sensitivity analysis | Sources and notes |
| --- | --- | --- | --- | --- |
| adr_r | Risk of acquired RIF resistance, for pan-susceptible TB treated with HRZE | 0.005 | 0.002-0.015 | ^27–29^ |
| adr_bpamz | Risk of acquired BDQ, PMD, or MFX resistance (same parameter applied to each drug independently), for pan-susceptible TB treated with BPaMZ | 0.002 | 0-0.01 | Chosen to have a combined probability of some resistance acquisition similar to that associated with HRZE, once applied to all 3 drugs (B,Pa,M). Also note ~1% acquisition of delamanid resistance in Study 213. |
| adr_mdr | Risk of acquired MFX resistance, for RIF-R or MDR TB treated with conventional MDR regimens | 0.04 | 0.005-0.08 | In PETTS (^30^ Table 2), the calculated proportion with acquired FQ resistance and a poor outcome among all MDR patients who didn't have FQ resistance initially, is 7%. In study 213 control arm (more modern regimens), 3.6% developed FQ-R. |
| adrfactor_other | Multiplicative increase in risk of acquired resistance, when resistance to one other key drug (INH-R, for HRZE; BDQ, PMD, or MFX-R, for BPaMZ) is present at the start of treatment | 8 | 3-15 | ^28^ |
| adrfactor_z | Multiplicative increase in risk of acquired BDQ, PMD, or MFX resistance, when resistance to PZA is present at the start of treatment | 1 | 1-3 | ^28^, and mechanism (not active where most bacilli are) |
| adrfactor_twodrugs | Multiplicative increase in risk of acquired BDQ, PMD, or MFX resistance, when resistance to the other two of these drugs is present at the start of treatment | 15 | 10-30 | ^28^ |
| adrfactor_partialmoxi | Multiplicative reduction if risk of acquired resistance when low-level MFX-R is present, relative to the risks above if high-level MFX-R is present | 0.67 | 0.5-1 | Assumed; chosen to be intermediate between full MFX and no MFX activity |

**Table S6: Calculated probabilities of acquired resistance, as a function of prescribed drugs that are active at baseline.** These follow from the parameters and assumptions described above. A sensitivity analysis increased the probabilities of acquired MFX-, BDQ-, and PMD-R by a factor of five for BPaMZ (to 1% each) and by a greater extent for subsets of BPaMZ (i.e. for use of BPaMZ when pre-existing resistance to one or more of BDQ, PMD, MFX, or PZA is present).

|  | RIF-R | INH-R | MFX-R | BDQ-R | PMD-R |
| --- | --- | --- | --- | --- | --- |
| HR(ZE) | 0.5% | 0.5% | NA | NA | NA |
| R(ZE) | 4.0% | NA | NA | NA | NA |
| H(ZE) | NA | 7.5% | NA | NA | NA |
| (ZE) | NA | NA | NA | NA | NA |
| MDR, MFX-S | NA | NA | 4.0% | NA | NA |
| MDR, MFX-low | NA | NA | 4.0% | NA | NA |
| MDR, MFX-high | NA | NA | NA | NA | NA |
| BPaMZ | NA | NA | 0.2% | 0.2% | 0.2% |
| BPaM | NA | NA | 0.2% | 0.2% | 0.2% |
| BPaZ | NA | NA | NA | 1.6% | 1.6% |
| BPamZ | NA | NA | 0.2% | 1.1% | 1.1% |
| BMZ | NA | NA | 1.6% | 1.6% | NA |
| PaMZ | NA | NA | 1.6% | NA | 1.6% |
| BPa | NA | NA | NA | 1.6% | 1.6% |
| BPam | NA | NA | 0.2% | 1.1% | 1.1% |
| BZ | NA | NA | NA | 3.0% | NA |
| BmZ | NA | NA | 1.6% | 2.0% | NA |
| BM | NA | NA | 1.6% | 1.6% | NA |
| PaZ | NA | NA | NA | NA | 3.0% |
| PamZ | NA | NA | 1.6% | NA | 2.0% |
| PaM | NA | NA | 1.6% | NA | 1.6% |
| MZ | NA | NA | 3.0% | NA | NA |
| B | NA | NA | NA | 83.3% | NA |
| Bm | NA | NA | 1.6% | 55.8% | NA |
| Pa | NA | NA | NA | NA | 83.3% |
| Pam | NA | NA | 1.6% | NA | 55.8% |
| M | NA | NA | 83.3% | NA | NA |
| Z | NA | NA | NA | NA | NA |
| mZ | NA | NA | 3.0% | NA | NA |
| m | NA | NA | 83.3% | NA | NA |
| none | NA | NA | NA | NA | NA |

*Simulating and reporting outcomes for a TB cohort*

For each implementation scenario, we simulated a minimum of 5,000 stochastic TB disease courses for each possible patient type in a cohort of representative TB cases; for those types that comprised more than 5% of the cohort, we simulated 50,000 possible courses. For each outcome reported, we then randomly drew from all possible disease courses, weighting according to the prevalence of each patient type within the setting-specific cohort, to create bootstrapped realizations of that outcome of interest among the entire cohort. Results are reported as mean and standard deviation across 50 bootstrapped realizations of the entire cohort of 100,000 TB cases. We verified that mean and standard deviation were stable across repeated runs of this size with different random seeds.

We evaluated TB cure in multiple ways, including: the proportion of all TB that was cured within N rounds of attempted treatment (including those with death before treatment, or initial loss to follow up, in the denominator); the proportion of individuals treated for TB (those who initiated some treatment regimen) who were cured; and the proportion of all people with TB cured within N months after TB onset. Additional outcomes reported include time with active TB or active drug-resistant TB, and months of TB treatment administered.

*Additional details of sensitivity analyses:*

Reduced BPaMZ efficacy: In this sensitivity analysis, we assumed that 5 months, rather than 4, were equivalent to 6 months of HRZE for pan-susceptible TB patients who completed the prescribed course of treatment. This increased relapse after 4 months of BPaMZ for pan-susceptible TB to 9.3% rather than 6.3% (still likely to fall within a clinical trial noninferiority margin). We also reduced the efficacy parameters for BPaM and BPaZ (proportion with culture conversion after 8 weeks) to the low ends of their respective uncertainty ranges as defined by Phase 2B trial data.

Improved MDR-TB standard of care: We reduced treatment duration to 12 months, scaled the treatment duration by a factor of 2 rather than 3 (relative to HRZE) in the regression model for treatment outcomes, and increased the parameter for probability of relapse and failure such that patients with MFX-S TB who complete MDR treatment would have a risk of relapse or failure experienced by RIF- and INH-susceptible TB after 6 months of HRZE. We maintained a constant relative risk of relapse and failure outcomes for MFX-R compared to MFX-S TB.

High risk of resistance acquisition with treated with BPaMZ: The risks of BDQ, PMD, and MFX resistance acquisition, when pan-susceptible TB is treated with BPaMZ, were increased to 1% each by increasing the parameter adr_bpamz to the high end of its sensitivity analysis range. In addition, we increased the parameters adr_factor_other and adr_factor_z to the high ends of their respective sensitivity analysis ranges.

**Supplemental results**

**Table S7: Additional measures of BPaMZ regimen impact in South African cohort**

| Scenario |  | Current care | RIF-R-only use of BPaMZ | Universal use of BPaMZ  (duration based on RIF-R result) |
| --- | --- | --- | --- | --- |
| Proportion cured after 1 treatment attempts, of all incident cases | Full cohort | 65.4 ± 0.1% | 65.9 ± 0.1% | 67 ± 0.2% |
|  | RS | 66.2 ± 0.2% | 66.5 ± 0.1% | 67 ± 0.2% |
|  | RR | 45 ± 0.7% | 50.3 ± 0.8% | 66.7 ± 0.6% |
|  | MFX-R | 54 ± 1.5% | 56.8 ± 1.4% | 58.8 ± 1.6% |
| Proportion cured after 2 treatment attempts, of all incident cases | Full cohort | 75.7 ± 0.1% | 76.2 ± 0.1% | 77.1 ± 0.2% |
|  | RS | 76.3 ± 0.1% | 76.6 ± 0.1% | 77.1 ± 0.2% |
|  | RR | 59.1 ± 0.7% | 64.5 ± 0.8% | 76.6 ± 0.7% |
|  | MFX-R | 66.2 ± 1.5% | 69 ± 1.3% | 71.2 ± 1.5% |
| Proportion cured within 36 months of TB onset | Full cohort | 67.7 ± 0.2% | 68.3 ± 0.1% | 69.8 ± 0.1% |
|  | RS | 68.6 ± 0.2% | 68.8 ± 0.2% | 69.8 ± 0.1% |
|  | RR | 46.4 ± 0.9% | 56.4 ± 0.8% | 68.9 ± 0.8% |
|  | MFX-R | 56 ± 1.5% | 61.4 ± 1.8% | 64.2 ± 1.9% |
| Proportion cured by first treatment course, of those who initiate treatment | Full cohort | 87.3 ± 0.1% | 87.6 ± 0.1% | 89.5 ± 0.1% |
|  | RS | 88.4 ± 0.1% | 88.5 ± 0.1% | 89.5 ± 0.1% |
|  | RR | 60 ± 0.9% | 67.1 ± 0.9% | 88.8 ± 0.6% |
|  | MFX-R | 72 ± 2% | 75.9 ± 1.7% | 78.7 ± 1.5% |
| Months with active TB, per incident TB case | Full cohort | 9.33 ± 0.04 | 9.21 ± 0.04 | 9 ± 0.04 |
|  | RS | 9.08 ± 0.03 | 9.03 ± 0.03 | 8.99 ± 0.04 |
|  | RR | 15.65 ± 0.45 | 13.86 ± 0.44 | 9.22 ± 0.2 |
|  | MFX-R | 13.05 ± 0.73 | 12.07 ± 0.78 | 11.36 ± 0.72 |
| Patient-months of treatment, per incident TB case | Full cohort | 5.44 ± 0.01 | 5.17 ± 0.01 | 3.47 ± 0.01 |
|  | RS | 5.15 ± 0.01 | 5.12 ± 0.01 | 3.44 ± 0 |
|  | RR | 12.91 ± 0.13 | 6.31 ± 0.08 | 4.34 ± 0.05 |
|  | MFX-R | 9.29 ± 0.3 | 5.86 ± 0.16 | 4.33 ± 0.1 |

**Figure S1: Dependence of acquired and propagated drug resistance on the baseline prevalence of BDQ resistance.**

**Fig S2: Dependence of BPaMZ impact on Xpert coverage and RIF-R prevalence, considering the alternative outcome of cure within two treatment attempts, among all incident TB cases**

**Figure S3: Higher prevalence of BPaMZ drug resistance (3x increased odds of MFX-R and of PZA-R)**

**Figure S4: Sensitivity analysis: Lower BPaMZ efficacy**

**Figure S5: Better standard of care for MDR-TB prior to BPaMZ introduction**

**Supplemental References**

1 WHO TB data repository. WHO. 2019. https://www.who.int/tb/country/data/download/en/ (accessed Jan 17, 2019).

2 World Health Organization, FIND. Technical report on critical concentrations for TB drug susceptibility testing of medicines used in the treatment of drug-resistant TB. World Health Organization, 2018 https://www.who.int/tb/publications/2018/WHO_technical_report_concentrations_TB_drug_susceptibility/en/ (accessed March 1, 2019).

3 Zignol M, Dean AS, Alikhanova N, *et al.* Population-based resistance of Mycobacterium tuberculosis isolates to pyrazinamide and fluoroquinolones: results from a multicountry surveillance project. *Lancet Infect Dis* 2016; **16**: 1185–92.

4 Villellas C, Coeck N, Meehan CJ, *et al.* Unexpected high prevalence of resistance-associated *Rv0678* variants in MDR-TB patients without documented prior use of clofazimine or bedaquiline. *J Antimicrob Chemother* 2016; **72**: dkw502.

5 National Institute for Communicable Diseases D of the NHLS. South African Tuberculosis Drug Resistance Survey 2012–14. http://www.nicd.ac.za/assets/files/K-12750 NICD National Survey Report_Dev_V11-LR.pdf (accessed April 3, 2019).

6 Alame-Emane AK, Xu P, Pierre-Audigier C, *et al.* Pyrazinamide resistance in &lt;I&gt;Mycobacterium tuberculosis&lt;/I&gt; arises after rifampicin and fluoroquinolone resistance. *Int J Tuberc Lung Dis* 2015; **19**: 679–84.

7 World Health Organization. Global tuberculosis report 2018. Geneva: World Health Organization, 2018 https://www.who.int/tb/publications/global_report/en/ (accessed Feb 28, 2019).

8 Rigouts L, Coeck N, Gumusboga M, *et al.* Specific gyrA gene mutations predict poor treatment outcome in MDR-TB. *J Antimicrob Chemother* 2016; **71**: 314–23.

9 Wood R, Lawn SD, Caldwell J, Kaplan R, Middelkoop K, Bekker L-G. Burden of New and Recurrent Tuberculosis in a Major South African City Stratified by Age and HIV-Status. *PLoS One* 2011; **6**: e25098.

10 Feldacker C, Tweya H, Keiser O, *et al.* Characteristics of adults and children diagnosed with tuberculosis in Lilongwe, Malawi: findings from an integrated HIV/TB clinic. *Trop Med Int Health* 2012; **17**: 1108–16.

11 Kunkel A, Abel zur Wiesch P, Nathavitharana RR, Marx FM, Jenkins HE, Cohen T. Smear positivity in paediatric and adult tuberculosis: systematic review and meta-analysis. *BMC Infect Dis* 2016; **16**: 282.

12 Subbaraman R, Nathavitharana RR, Satyanarayana S, *et al.* The Tuberculosis Cascade of Care in India’s Public Sector: A Systematic Review and Meta-analysis. *PLoS Med* 2016; **13**: e1002149.

13 Tiemersma EW, van der Werf MJ, Borgdorff MW, Williams BG, Nagelkerke NJD. Natural History of Tuberculosis: Duration and Fatality of Untreated Pulmonary Tuberculosis in HIV Negative Patients: A Systematic Review. *PLoS One* 2011; **6**: e17601.

14 Dorman SE, Schumacher SG, Alland D, *et al.* Xpert MTB/RIF Ultra for detection of Mycobacterium tuberculosis and rifampicin resistance: a prospective multicentre diagnostic accuracy study. *Lancet Infect Dis* 2018; **18**: 76–84.

15 Kruk ME, Schwalbe NR, Aguiar CA. Timing of default from tuberculosis treatment: a systematic review. *Trop Med Int Heal TM IH* 2008; **13**: 703–12.

16 Marx FM, Dunbar R, Enarson DA, *et al.* The Temporal Dynamics of Relapse and Reinfection Tuberculosis After Successful Treatment: A Retrospective Cohort Study. *Clin Infect Dis* 2014; **58**: 1676–83.

17 Gegia M, Winters N, Benedetti A, van Soolingen D, Menzies D. Treatment of isoniazid-resistant tuberculosis with first-line drugs: a systematic review and meta-analysis. *Lancet Infect Dis* 2017; **17**: 223–34.

18 Wallis RS, Wang C, Meyer D, Thomas N. Month 2 Culture Status and Treatment Duration as Predictors of Tuberculosis Relapse Risk in a Meta-Regression Model. *PLoS One* 2013; **8**: e71116.

19 Wallis RS, Peppard T, Hermann D. Month 2 culture status and treatment duration as predictors of recurrence in pulmonary tuberculosis: model validation and update. *PLoS One* 2015; **10**: e0125403.

20 Dawson R, Harris K, Conradie A, *et al.* Efficacy of Bedaquiline, Pretomanid, Moxifloxacin &amp; PZA (BPaMZ) Against DS- &amp; MDR-TB. CROI. 2017. https://www.tballiance.org/ (accessed Feb 11, 2019).

21 Li S-Y, Tasneen R, Tyagi S, *et al.* Bactericidal and Sterilizing Activity of a Novel Regimen with Bedaquiline, Pretomanid, Moxifloxacin, and Pyrazinamide in a Murine Model of Tuberculosis. *Antimicrob Agents Chemother* 2017; **61**: e00913-17.

22 Dawson R, Diacon AH, Everitt D, *et al.* Efficiency and safety of the combination of moxifloxacin, pretomanid (PA-824), and pyrazinamide during the first 8 weeks of antituberculosis treatment: a phase 2b, open-label, partly randomised trial in patients with drug-susceptible or drug-resistant pul. *Lancet* 2015; **385**: 1738–47.

23 WHO Treatment Guidelines for Drug-Resistant Tuberculosis, 2016 Update. Geneva: World Health Organization, 2016 http://www.ncbi.nlm.nih.gov/books/NBK390455/.

24 Ahuja SD, Ashkin D, Avendano M, *et al.* Multidrug Resistant Pulmonary Tuberculosis Treatment Regimens and Patient Outcomes: An Individual Patient Data Meta-analysis of 9,153 Patients. *PLoS Med* 2012; **9**: e1001300.

25 Fox W, Ellard GA, Mitchison DA. Studies on the treatment of tuberculosis undertaken by the British Medical Research Council Tuberculosis Units, 1946–1986, with relevant subsequent publications. https://www.ingentaconnect.com/contentone/iuatld/ijtld/1999/00000003/A00210s2/art00001# (accessed Jan 4, 2019).

26 Ahmad N, Ahuja SD, Akkerman OW, *et al.* Treatment correlates of successful outcomes in pulmonary multidrug-resistant tuberculosis: an individual patient data meta-analysis. *Lancet* 2018; **392**: 821–34.

27 Menzies D, Benedetti A, Paydar A, *et al.* Effect of duration and intermittency of rifampin on tuberculosis treatment outcomes: a systematic review and meta-analysis. *PLoS Med* 2009; **6**: e1000146.

28 Lew W, Pai M, Oxlade O, Martin D, Menzies D. Initial drug resistance and tuberculosis treatment outcomes: systematic review and meta-analysis. *Ann Intern Med* 2008; **149**: 123–34.

29 Rockwood N, Sirgel F, Streicher E, Warren R, Meintjes G, Wilkinson RJ. Low Frequency of Acquired Isoniazid and Rifampicin Resistance in Rifampicin-Susceptible Pulmonary Tuberculosis in a Setting of High HIV-1 Infection and Tuberculosis Coprevalence. *J Infect Dis* 2017; **216**: 632–40.

30 Cegielski JP, Kurbatova E, van der Walt M, *et al.* Multidrug-resistant tuberculosis treatment outcomes in relation to treatment and initial versus acquired second-line drug resistance. *Clin Infect Dis* 2016; **62**: 418–30.
